# Disordered Eating in Asian American Women: Sociocultural and Culture-Specific Predictors

**DOI:** 10.3389/fpsyg.2019.01950

**Published:** 2019-09-04

**Authors:** Liya M. Akoury, Cortney S. Warren, Kristen M. Culbert

**Affiliations:** ^1^Department of Psychology, University of Nevada, Las Vegas, Las Vegas, NV, United States; ^2^Choose Honesty, LLC, Las Vegas, NV, United States

**Keywords:** disordered eating, Asian American women, sociocultural model, ethnic identity, biculturalism, acculturative stress, acculturation

## Abstract

Asian American women demonstrate higher rates of disordered eating than other women of color and comparable rates to European American women. Research suggests that leading sociocultural predictors, namely, pressures for thinness and thin-ideal internalization, are predictive of disordered eating in Asian American women; however, no known studies have tested the intersection of sociocultural and culture-specific variables (e.g., ethnic identity, biculturalism, and acculturative stress) to further elucidate disordered eating risk in this vulnerable, understudied group. Accordingly, this project used path analysis to simultaneously examine the role of sociocultural and culture-specific effects on disordered eating in Asian American college women (*N* = 430). Self-report measures assessing disordered eating, sociocultural (pressures for thinness, thin-ideal internalization), and culture-specific (ethnic identity, biculturalism, acculturative stress) variables revealed that a number of sociocultural and culture-specific factors are predictive of disordered eating. Consistent with prior research, heightened perceived pressures for thinness and thin-ideal internalization were predictive of disordered eating, and thin-ideal internalization partially mediated the relationship between pressures for thinness and disordered eating. Acculturative stress predicted disordered eating and fully accounted for the inverse relationship between biculturalism and disordered eating. Overall, findings highlighted the salience of sociocultural predictors for disordered eating in Asian American women and identified biculturalism and acculturative stress as culture-specific contributors that may uniquely impact vulnerability to disordered eating in Asian American women. Thus, the combined consideration of sociocultural and culture-specific factors may be important in disordered eating research and in the development of individualized treatment plans for Asian American women.

## Introduction

Risk for disordered eating is multifaceted, involving the intersection of a variety of biological (e.g., genetic vulnerability), psychological (e.g., personality), sociocultural (e.g., internalization of the thin ideal), and culture-specific (e.g., ethnic identity) factors. While a complex interplay among multiple factors is expected to constitute disordered eating risk (Keel and Forney, 2013; Culbert et al., 2015), a number of gaps remain in current etiologic models. In particular, most studies have been conducted in European or European American women. Furthermore, the extent to which sociocultural (e.g., internalization of the thin ideal) and culture-specific (e.g., ethnic identity) factors contribute, and possibly intersect, to alter disordered eating risk in ethnic minority groups is largely unknown. While sociocultural factors are well-established risk factors for disordered eating in European American women (Keel and Forney, 2013; Culbert et al., 2015), evidence for a role of sociocultural factors *or* culture-specific factors in disordered eating in ethnic minority women is relatively sparse (Soh and Walter, 2013). Notably, culture-specific factors may influence disordered eating directly (i.e., change in factor results in change in disordered eating), indirectly (i.e., change in factor results in changes in other factors, which directly predict disordered eating), or both. As such, comprehensive examination of multi-faceted disordered eating modeling is warranted.

One ethnic minority group of particular interest is Asian Americans, as they are the fastest growing ethnic minority group in the United States (Mackun et al., 2011), yet are comparatively understudied in disordered eating research (Soh and Walter, 2013). Mental health problems, including disordered eating, are under-diagnosed among Asian Americans, due to mental health professionals’ assumptions that they are well-adjusted (i.e., the “model minority” stereotype; Osajima, 2005; Lee et al., 2009). However, in contrast to the model minority stereotype, recent research suggests that Asian American women endorse comparable levels of disordered eating to European American women (Franko et al., 2007; Gillen and Lefkowitz, 2012).

While incidence of disordered eating is comparable between Asian American and European American women, risk for disordered eating in Asian American women may be unique from both European American and Asian (non-diasporan) women in terms of sociocultural and culture-specific influences. The primary sociocultural model of disordered eating posits that women in Westernized cultures are inundated with messages that beauty is equated with the attainment of a thin body (Scharrer, 2013). Notably, Asian and Asian American cultures endorse a similar heightened value for the thin ideal as mainstream Western culture (e.g., Luo et al., 2005). These cultural values that promote the idealization of thinness can heighten risk for disordered eating. Specifically, women experience pressures to maintain or achieve thinness through multiple sources, such as the media (e.g., magazine advertisements), family members (e.g., parents encouraging weight loss), and peers (e.g., weight-related teasing). Some women ultimately internalize (e.g., “buy into”) this socially construed thin ideal as personally important to achieve, despite its general unattainability. Elevated levels of perceived pressures for thinness and thin-ideal internalization have been shown to prospectively predict the development of disordered eating (for a review, see Culbert et al., 2015), and thin-ideal internalization has been identified as a mediator of the relationship between pressures for thinness and disordered eating (Cafri et al., 2005; Moreno-Domínguez et al., 2019). This sociocultural model has been extensively studied in European American and European women (Soh and Walter, 2013), and emerging research has shown that it is also applicable to disordered eating risk in Asian (Lee and Lee, 2000) and Asian American women (Nouri et al., 2011; Smart and Tsong, 2014).

Despite the salience of the sociocultural model in Asian American women, not all women who endorse heightened pressures for thinness and/or thin-ideal internalization develop disordered eating. Additionally, the examination of sociocultural factors in isolation, as opposed to considering the integration of culturally relevant variables, presumes and retrofits minority individuals into etiologic models that have been largely constructed from European American samples. As such, identifying culture-specific factors that uniquely contribute to and/or intersect with sociocultural factors to predict individual differences in vulnerability to disordered eating in Asian American women is important. These factors, relating to Asian Americans’ experiences as an ethnic minority group in the United States, may differentiate their disordered eating etiology from both Asian and European American women. For example, data suggest that *ethnic identity* (i.e., sense of belonging to one’s ethnic/cultural group; Phinney, 1990) buffers the relationship between sociocultural influences (i.e., pressures for thinness, thin-ideal internalization) and disordered eating in ethnic minority women, including Asian Americans (Rakhkovskaya and Warren, 2014, 2016). Specifically, the predictive effects of pressures for thinness and thin-ideal internalization on disordered eating were more pronounced for women who reported low ethnic identity, whereas a strong ethnic identity appeared to be protective against sociocultural influences on elevated disordered eating symptoms (Rakhkovskaya and Warren, 2014, 2016). These findings suggest that Asian American women with low ethnic identity may be particularly vulnerable to cultural messages about the idealization of thinness and the subsequent development of disordered eating.

Another culture-specific factor of relevance to understanding disordered eating in Asian American women is acculturation-related experiences within the United States. Prominent acculturation theory suggests that acculturation is multidimensional, such that individuals may identify predominantly with their acquired culture (*assimilation*), their native culture (*separation*), both their acquired and native culture (*integration or biculturalism*), or neither (*marginalization*; Berry, 2005; Berry et al., 2006). Among these dimensions of acculturation, biculturalism is associated with the best mental health outcomes whereas marginalization is associated with the worst (Berry, 2005; Berry et al., 2006). Unfortunately, no known study has examined the relationship between biculturalism and disordered eating. Instead, studies have taken a unidimensional approach by focusing on assimilation (rather than biculturalism), which has produced inconclusive results (e.g., Jackson et al., 2006; Jennings et al., 2006; Aruguete et al., 2007). Nevertheless, given negative associations between low biculturalism and mental health difficulties (Berry, 2005; Berry et al., 2006), low biculturalism may also predict higher levels of disordered eating. Additionally, the pathways between low biculturalism and elevated levels of disordered eating may be indirect, via ethnic identity and *acculturative stress* (i.e., the aggregate physical, biological, social, cultural, and psychological difficulties that individuals face as they encounter a new culture; Oh et al., 2002; Berry, 2006). Biculturalism is positively associated with ethnic identity (e.g., Roysircar-Sodowsky and Maestas, 2000) *and* negatively associated with acculturative stress (e.g., Oh et al., 2002). A small body of research also suggests that higher levels of acculturative stress predict greater mental health concerns (e.g., Oh et al., 2002), including disordered eating (Reddy and Crowther, 2007). Taken together, biculturalism may be an indirect predictor of disordered eating such that (1) lower biculturalism is associated with *lower* ethnic identity, which may increase vulnerability to higher disordered eating; and (2) the negative relationship between biculturalism and disordered eating may be fully or largely mediated by acculturated stress, as has been shown for other health outcomes (e.g., Oh et al., 2002).

**FIGURE 1:**
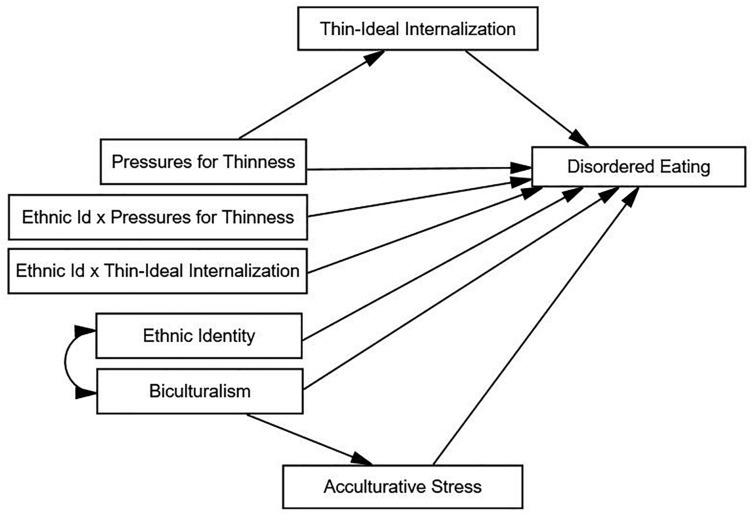
Hypothesized model for disordered eating in Asian American women. Ethnic Id × Pressures for Thinness, ethnic identity by pressures for thinness interaction term; Ethnic Id × Thin-Ideal Internalization, ethnic identity by thin-ideal internalization interaction term.

This study aimed to build upon prior findings by simultaneously examining sociocultural (i.e., pressures for thinness; thin-ideal internalization) and culture-specific (i.e., ethnic identity; biculturalism; acculturative stress) predictors of disordered eating in Asian American women *and* considering the possible interplay between variables (Figure 1). Four primary aims/hypotheses were examined within the model:

Aim 1: Test the sociocultural model for disordered eating. In accordance with prior findings, including those in Asian American women (Nouri et al., 2011; Smart and Tsong, 2014), it was hypothesized that the predictive effects of pressures for thinness on disordered eating will be mediated by thin-ideal internalization.
Aim 2: Test ethnic identity as a buffer (i.e., moderator) against sociocultural effects on disordered eating. Similar to prior research (Rakhkovskaya and Warren, 2014, 2016), higher ethnic identity was expected to attenuate relationships between sociocultural variables (i.e., pressures for thinness and thin-ideal internalization) and disordered eating.
Aim 3: Test the relationship between ethnic identity and biculturalism in Asian American women. Biculturalism was expected to show a bi-directional positive association with ethnic identity, as has been shown in prior work (e.g., Roysircar-Sodowsky and Maestas, 2000).
Aim 4: Test the interrelationships between biculturalism, acculturative stress, and disordered eating. Extrapolating from the mediation-based effects that have been found for other health outcomes (Oh et al., 2002), lower biculturalism was expected to be predictive of higher levels of disordered eating, and this inverse relationship was expected to be mediated by heightened levels of acculturative stress.

## Materials and Methods

### Participants

Asian American college women participated in an online study on eating, personality, and mood. Participants were simultaneously recruited via: (1) the university psychology department’s online subject pool (i.e., Sona Systems; *n* = 250, 58.14% of sample) and (2) advertisements in student organizations (e.g., list serves) and flyers posted on campus and in the surrounding community (*n* = 180; 41.86% of sample). A total of 549 participants signed up for the study, of which, 119 were removed due to a failure to meet inclusion criteria. Specifically, participants who did not identify as Asian or Asian American, were over 40 years of age (*n* = 15), completed <50% of the study (*n* = 87), or provided questionable data (e.g., responded carelessly as evidenced by identical responses across all items for entire questionnaires or spending <20 min completing the extensive survey) (*n* = 17) were removed. This yielded a final sample of 430 participants (ages 18–40 years; *M* = 20.64; *SD* = 2.61).

Table 1 summarizes sample descriptives. With regard to race, 73.33% (*n* = 311) of the sample identified as Asian American only and 27.67% (*n* = 119) identified as multiracial (i.e., Asian American and at least one other race). One-way analysis of variance (ANOVA) showed no differences on any study variables between participants who identified as Asian American only (i.e., uniracial Asian Americans) and participants who identified as multiracial.

**TABLE 1:** Descriptive data on BMI, race, ethnicity, and generational status.

**Variable**	***n***	**% Total sample**
**BMI category**		
Underweight (<20 kg/m^2^)	86	20.00
Average weight (20–25 kg/m^2^)	206	47.91
Overweight (25–30 kg/m^2^)	68	15.81
Obese (>30 kg/m^2^)	33	7.67
Unknown (missing data)	37	8.60
**Race**		
Asian American only	311	73.33
Asian American and other race(s)	119	27.67
**Racial breakdown^†^**		
East Asian, Southeast Asian, or Asian American	365	84.88
Native Hawaiian or Other Pacific Islander	61	14.19
South Asian or South Asian American	59	13.72
European American or White	39	9.07
Latina or Latina American	15	3.49
African American or Black	7	1.63
American Indian or Alaskan Native	3	0.70
Middle Eastern or Middle Eastern American	2	0.47
Other	1	0.23
**Ethnicity^†^**		
Filipino	233	54.19
Chinese	89	20.70
Japanese	48	11.16
Vietnamese	34	7.91
Korean	32	7.44
Other Asian American	25	5.81
South Asian (Indian, Pakistani, Sri Lankan)	17	3.95
Thai	11	2.56
Taiwanese	10	2.33
Cambodian	8	1.86
Hawaiian/Pacific Islander	7	1.63
Laotian	6	1.40
**Generational status**		
International student (non-immigrant)	7	1.63
First generation	98	22.79
Second generation	235	54.65
Third generation	20	4.65
Fourth generation	21	4.88
Fifth of greater generation	14	3.26

*BMI, body mass index. **^†^**Multi-choice variables, such that percentages exceed 100% (i.e., participants could select all races and ethnicities that apply).*

Participants endorsed 14 different Asian ethnicities (Table 1), but were predominantly Filipino (39.5%; *n* = 233), Chinese (20.70%; *n* = 89), and multi-ethnic Asian American (e.g., mixed Japanese, Chinese, and Filipino ancestry; 21.63%; *n* = 93). Most participants (73.49%; *n* = 316) were born in the United States, and the majority of the sample was first-generation (22.79%; *n* = 98) or second-generation (54.65%; *n* = 235) Americans. One-way ANOVAs tested whether participants differed by generational status on any of the study variables and indicated that first-generation participants reported significantly lower pressures for thinness than second-generation participants [*F*(2,403) = 3.49, *p* = 0.03; pairwise *d* = 0.29], and second-generation participants had significantly *lower* biculturalism than participants who were third generation or higher [*F*(2,396) = 3.25, *p* = 0.04; pairwise *d* = 0.42]. As such, generational status was added as a covariate in analyses.

### Procedure

The university’s Institutional Review Board (IRB Protocol No. 788816-3) approved this study protocol and participants completed informed consent electronically prior to the completion of the surveys. To minimize missing data, the survey software reminded (but did not require) participants to respond to missed items before proceeding. Subject pool participants received credits for participation, whereas participants recruited *via* campus/community advertisements were offered a $10 online Amazon.com gift card.

### Measures

#### Disordered Eating

The global score of the Eating Disorder Examination Questionnaire (EDE-Q; Fairburn and Beglin, 1994) assessed overall levels of disordered eating symptoms in terms of (1) dietary restraint (i.e., attempts to limit or avoid food consumption), (2) eating concerns (i.e., preoccupation with food, secretiveness, and guilt about eating), (3) shape concerns (i.e., preoccupation with body shape), and (4) weight concerns (i.e., preoccupation with weight and weight loss). Items (e.g., have you had a definite desire to have a totally flat stomach? How dissatisfied have you been with your shape)? are rated over the past 28 days on a seven-point scale (e.g., “No days” to “Every day” or “Not at all” to “Markedly”), with higher scores indicating higher levels of disordered eating symptoms (Fairburn and Beglin, 1994). The EDE-Q global score has demonstrated good psychometric properties in prior studies of adult women (Mond et al., 2006), including Asian American college women (Rakhkovskaya and Warren, 2016) and Japanese college women (α = 0.74–0.89; Nakai et al., 2014); internal consistency was also excellent in this sample (α = 0.93).

#### Sociocultural Influences

Selected subscales of the Sociocultural Attitudes Toward Appearance Questionnaire-4 (SATAQ-4; Schaefer et al., 2014) assessed sociocultural pressures and attitudes regarding the thin ideal. Specifically, this study focused on the thin-ideal internalization (i.e., endorsement of the mainstream beauty ideal as thin) and pressures for thinness subscales: media pressure (i.e., pressure from mainstream media to adhere to thin or athletic ideal), family pressure (i.e., pressure from family members to adhere to thin or athletic ideal), and peer pressure (i.e., pressure from peers to adhere to thin or athletic ideal). Items (e.g., “My peers encourage me to get thinner”) are rated on a five-point Likert scale ranging from “Definitely Disagree” to “Definitely Agree”; thus, higher scores indicate higher thin-ideal pressure or internalization. This study combined the media, peer, and family pressures subscales into a combined pressures for thinness scale, similar to prior research (Rakhkovskaya and Warren, 2016; Yamamiya et al., 2016). These SATAQ-4 subscales have demonstrated good to excellent internal consistency in prior studies of ethnic minority women (α = 0.86–0.95; Llorente et al., 2014; Schaefer et al., 2014) and in this sample of Asian American women (α = 0.80 for thin-ideal internalization; α = 0.89 for combined pressures for thinness).

#### Ethnic Identity

The ethnic identity subscale of the Multigroup Ethnic Identity Measure (MEIM-EI; Phinney, 1992) assessed ethnic identity. The MEIM assesses ethnic attitudes and behaviors across two subscales: ethnic identity (i.e., a sense of belonging to one’s ethnic or cultural group) and other-group orientation (i.e., a sense of belonging to the majority culture or group – European American). In accordance with extant research on ethnic identity and disordered eating (Rakhkovskaya and Warren, 2014, 2016), this study solely used the ethnic identity subscale to assess participants’ attachment to their ethnic or subethnic group (e.g., Asian, Korean). Ethnic identity examples items include “I am active in organizations or social groups that include mostly members of my own ethnic group” and “I am happy that I am a member of the group I belong to.” Items are rated on a four-point Likert scale, ranging from “Strongly Disagree” to “Strongly Agree,” with higher scores indicating stronger ethnic identity. The MEIM demonstrated strong psychometric properties in numerous demographic groups, including Asian American college students (Lee and Yoo, 2004). The ethnic identity subscale had good internal consistency in prior research (α = 0.81–0.92; Ponterotto et al., 2003) and in this sample (α = 0.75).

#### Biculturalism

The Bicultural Identity Integration Scale (BIIS; Benet-Martínez and Haritatos, 2005) assessed biculturalism. The BIIS is an eight-item self-report scale with two subscales. The cultural conflict subscale measures an affective perception that one’s native and majority cultures clash with one another, which is indicative of a *lack of* cultural integration or *lack of* biculturalism (Berry, 2005; Berry et al., 2006). The cultural distance subscale measures an affective perception that one’s native and majority cultures are distant. Items are rated on a five-point Likert scale, ranging from “Definitely Not True” to “Definitely True.” In accordance with prior research (Thomas et al., 2010), only the cultural conflict subscale was used to assess biculturalism; example items include: “I feel caught between the Asian and American cultures” and “I feel like someone moving between two cultures.” *Lower* cultural conflict scores indicate *higher* levels of biculturalism (Benet-Martínez and Haritatos, 2005); however, to ease interpretation, the cultural conflict scale was reverse-scored, so that *higher* scores would indicate *higher* levels of biculturalism (i.e., higher cultural integration). The BIIS cultural conflict subscale demonstrated acceptable internal consistency in prior research (e.g., α = 0.74; Benet-Martínez and Haritatos, 2005) and in this sample (α = 0.86).

#### Acculturative Stress

The Social Attitudinal Familial and Environmental Acculturative Stress Scale (SAFE; Mena et al., 1987) assessed acculturative stress. The SAFE measures acculturative stress across social, attitudinal, familial, and environmental contexts (e.g., “People look down upon me when I practice my Asian customs”; “My family members and I have different expectations about my future”). Items are rated on a six-point Likert scale, ranging from “Not Stressful” (1) to “Extremely Stressful” (5), with an additional option of “Have Not Experienced” (0). Higher scores indicate higher levels of acculturative stress. Internal consistency for Asian American students was good in prior research (α = 0.89–0.91; Mena et al., 1987; Claudat et al., 2016) and in this sample (α = 0.90).

#### Covariates

We assessed body mass index (BMI) and generational status and included both as covariates in this study, given prior research demonstrating that BMI (e.g., Rakhkovskaya and Warren, 2014, 2016) and generational status (Tsong and Smart, 2016) are associated with disordered eating and sociocultural factors, like pressures for thinness. We collected height and weight via self-report and calculated BMI (kg/m^2^). Self-reported height and weight correlate highly with laboratory measurements and, thus, serve as a reasonable approximation of true BMI (McAdams et al., 2007).

### Statistical Analyses and Analytic Plan

Statistical Package for Social Sciences (SPSS) version 20 was used for descriptive analyses. AMOS SPSS version 25 was used to conduct path analysis using the maximum-likelihood estimation method, per recommendations (Bentler, 2006).

#### Data Preparation

We prorated scales that contained 10 or more items for participants missing 10% or fewer of items. We coded scores as missing for participants missing items on scales that contain <10 items. BMI was inversely transformed to adjust for positive skew, and generational status was dummy coded (first-generation Americans as the reference group). Prior to conducting path analysis (see below), we standardized all continuous variables, so that path estimates would reflect standardized coefficients. Two interaction term variables were created to allow for the examination of the hypothesized moderation effects. Specifically, we multiplied ethnic identity by pressures for thinness (i.e., ethnic identity × pressures for thinness interaction) and by thin-ideal internalization (i.e., ethnic identity × thin-ideal internalization interaction). Path analysis handled missing data using AMOS maximum-likelihood imputation algorithm.

#### Correlations

Pearson correlations tested initial associations between all predictor and outcome variables.

#### Path Analysis

Path analysis tested the primary study hypotheses, as path analysis can analyze complex relational models and can empirically test how well the hypothesized effects fit the data (Streiner, 2005). A series of unconstrained and constrained nested models were fit (i.e., differing in one or more parameters). We first fit the overall unconstrained model that included sociocultural and culture-specific variables as predictors of disordered eating as well as BMI and generational status as covariates (Figure 1). We then directly tested whether thin-ideal internalization fully mediates the relationship between pressures for thinness and disordered eating by comparing fit of the unconstrained sociocultural model to a submodel that constrained the direct path (i.e., set to zero, indicating this path had no direct effect) from pressures for thinness to disordered eating (Aim 1). Next, we evaluated whether ethnic identify moderates sociocultural effects on disordered eating based on the significance of the “ethnic identity × pressures for thinness” and “ethnic identity × thin-ideal internalization” path estimates (Aim 2). We also tested the bidirectional relationship between ethnic identity and biculturalism (Aim 3). Finally, we determined whether acculturative stress fully mediates the relationship between biculturalism and disordered eating, by comparing the fit of the unconstrained comprehensive model to a submodel that constrained the direct path from biculturalism to disordered eating (Aim 4).

Several strategies evaluated study hypotheses. Specifically, we examined the significance of the standardized path estimates and made model fit comparisons via chi-squared difference tests between the full/unconstrained and nested models (Bryant and Satorra, 2012). The chi-squared value from the least restrictive model was subtracted from the chi-squared value for the more restrictive model. The significance of the resulting chi-square difference value (with the remaining degrees of freedom) indicated model fit, with a significant chi-squared value suggestive of worse fit of the nested model. We also used the comparative fit index (CFI; Bentler, 1990), root-mean-square error of approximation (RMSEA; Steiger and Lind, 1980), and Akaike’s information criterion (AIC; Akaike, 1987) to evaluate model fit. A CFI >0.95 and a RMSEA <0.08 indicate good fit, and models with a smaller AIC are preferred (Hu and Bentler, 1999).

Importantly, if a model provided poor fit to the data or negligible hypothesized effects, we considered model adjustments to improve model fit. Namely, non-significant predictors were dropped, if such adjustments were justified. Per recommendations for path analyses (Mueller and Hancock, 2008), no further trimming was performed to minimize the number of statistical tests and to ensure that only theoretically sound alternative models were examined.

## Results

There was adequate variability in disordered eating and all predictor variables (Table 1). Approximately 12% (*n* = 52) of the sample scored above the EDE-Q clinical mean, indicating that a substantial proportion of participants scored in a clinically significant range (Welch et al., 2011). Participants also spanned a range of BMI categories, but nearly half of the sample fell within the “average weight” range (Table 1). Mean levels of disordered eating [*F*(3,392) = 22.88, *p* < 0.01, *d*s = 0.21–1.26] and pressures for thinness [*F*(3,392) = 17.67, *p* < 0.01, *d*s = 0.27–1.38] significantly differed across BMI categories.

### Correlations

Several notable associations were detected between study variables (Table 2). As expected, disordered eating was significantly positively correlated with all sociocultural variables (i.e., perceived pressures for thinness; thin-ideal internalization; *r*s = 0.62–0.65; *p*s < 0.01). BMI was significantly positively correlated with disordered eating and perceived pressures for thinness (*r*s = 0.36 and 0.35, respectively; *p*s < 0.01), but not thin-ideal internalization or any of the culture-specific variables (e.g., biculturalism). Ethnic identity was not significantly correlated with disordered eating or other study variables, but biculturalism and acculturative stress were inversely correlated (*r* = −0.35, *p* < 0.01) and showed significant associations with disordered eating, pressures for thinness, and thin-ideal internalization. Specifically, biculturalism showed small inverse associations with pressures for thinness, thin-ideal internalization, and disordered eating (*r*s = −0.16 to −0.23; *p*s < 0.01), and acculturative stress showed positive associations, pressures for thinness, thin-ideal internalization (*r*s = 0.13–0.23; *p*s < 0.05), and disordered eating (*r* = 0.21, *p* < 0.01).

**TABLE 2:** Pearson’s correlations and descriptive statistics for study variables.

	**2**	**3**	**4**	**5**	**6**	**7**	**8**	**Mean**	***SD***	**Range**
1. Disordered eating	**0.62^∗∗^**	**0.65^∗∗^**	–0.05	**−0.17^∗∗^**	**0.21^∗∗^**	**0.36^∗∗^**	0.08	2.25	1.34	0.00–5.45
2. Pressures for thinness	–	**0.55^∗∗^**	0.02	**−0.23^∗∗^**	**0.23^∗∗^**	**0.35^∗∗^**	–0.03	3.03	0.89	1.00–5.00
3. Thin-ideal internalization		–	–0.01	**−0.16^∗∗^**	**0.13^∗^**	0.08	–0.06	3.84	0.65	1.73–5.00
4. Ethnic identity			–	−0.05	−0.04	0.01	–0.05	2.85	0.48	1.38–4.00
5. Biculturalism				–	**−0.35^∗∗^**	–0.03	**−0.14^∗∗^**	3.40	1.16	1.00–5.00
6. Acculturative stress					–	0.03	0.01	52.39	14.13	24.00–99.00
7. BMI						–	**0.17^∗∗^**	23.23	4.55	15.34–44.91
8. Age							–	20.64	2.61	18.00–40.00

*^∗∗^*p* < 0.01; ^∗^*p* < 0.05. Bolded text indicates a significant correlation. BMI, body mass index; SD, standard deviation.*

### Path Analyses

#### Adjustments for Model Identification

Initial inspection of the proposed models by AMOS indicated that they were under-identified (i.e., models could not be tested by AMOS without imposing additional constraints). Thus, we applied additional constraints to attain model identification. First, we set the means of standardized variables (i.e., all variables, except ethnic identity by sociocultural predictors interaction terms) to zero, in accordance with recommendations (Gunzler and Morris, 2015). Constraining standardized variables’ means to zero would not unduly influence the models, as these means were already known to equal zero by virtue of standardization. This approach increased degrees of freedom to help achieve model over-identification without losing model generality. Second, we set the regression weights of all error terms to 1, per recommendations (Arbuckle, 2016). This further helped achieve model identification through assigning the error terms a unit of measurement, and this approach likewise did not unduly influence the models, as standardized regression terms are unaffected by choice of identification constraints (Arbuckle, 2016). The aforementioned model modifications did not influence the overall visual depiction of the proposed model (shown in Figure 1).

#### Model Fit

**FIGURE 2:**
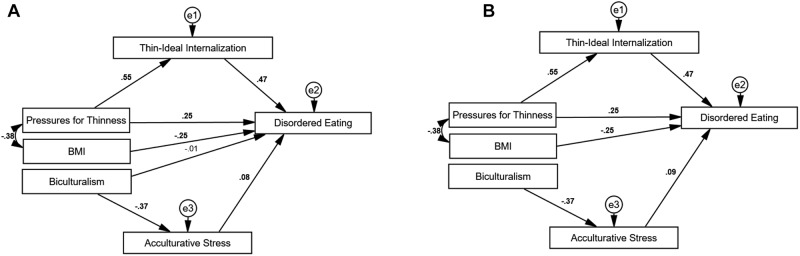
Model 1.1 and Model 3: Revised unconstrained and best-fitting models for disordered eating. **(A)** Model 1.1: Revised unconstrained model. **(B)** Model 3: Best-fitting model. BMI, body mass index; e1, e2, and e3, error terms. Statistically significant path estimates are in bold (*p* < 0.05).

Table 3 presents model fit statistics and interpretations. The initial unconstrained model demonstrated poor overall fit (see Model 1); thus, model modifications were made to produce a simplified model that is more representative of the data. Specifically, the revised model (Model 1.1) dropped four non-significant paths (i.e., generational status, ethnic identity, ethnic identity × pressures for thinness, and ethnic identity × thin-ideal internalization). The biculturalism to disordered eating path was retained, despite being non-significant, to allow for the subsequent test of acculturative stress mediation effects, as mediation is possible in the absence of direct effects (Hayes, 2017). This revised model (Model 1.1) provided good fit and served as the base model^1^ (Figure 2A).

**TABLE 3:** Path analysis model comparisons and goodness-of-fit statistics.

**Model**	**χ^2^ (*df*)**	**Δχ^2^ (*df*)**	***p***	**CFI**	**RMSEA**	**AIC**	**Interpretation**
1. Unconstrained model	245.84 (38)	–	–	0.75	0.11	299.84	Poor fit
1.1 Revised unconstrained model: drop generational status and ethnic identity	46.16 (13)	–	–	0.95	0.08	74.16	Good fit
2. Revised constrained model: constrain pressure for thinness → DE (test full mediation)	80.16 (14)	34.00 (1)	<0.01	0.90	0.11	106.16	Model 1.1 better fit than Model 2 (partial mediation only)
3. **Revised constrained model: constrain biculturalism → DE (test full mediation)**	**46.21 (14)**	**0.05 (1)**	**0.82**	**0.95**	**0.07**	**72.31**	**Model 3 better fit than Model 1.1 (full mediation)**

*DE, disordered eating. χ^2^, chi-squared statistic; *df*, degrees of freedom; CFI, comparative fit index; RMSEA, root-mean-square error of approximation; AIC, Akaike’s information criterion; Δ*χ*^2^, chi-squared difference statistic; *p*, *p*-value of chi-squared difference statistic. For model comparisons, least restrictive models are subtracted from most restrictive models, and a *non-significant* Δ*χ*^2^ indicates that the *least* restrictive model has better fit. Bolded text indicates best-fitting model.*

A constrained model (Model 2), where the pressures for thinness path were set to zero, determined if thin-ideal internalization fully mediates the relationship between pressures for thinness and disordered eating. This model demonstrated adequate overall fit, yet the chi-square difference test indicated that Model 1.1 continued to be best-fitting. Despite a lack of evidence for full mediation, significant positive relationships among pressures for thinness, thin-ideal internalization, and disordered eating suggest that thin-ideal internalization likely serves as a partial mediator.

Next, the biculturalism path was set to zero to test whether acculturative stress fully mediates the biculturalism-disordered eating relationship (Model 3), which resulted in improved fit over Model 1.1 (i.e., non-significant chi-square difference test, lower AIC), suggesting that the relationship between biculturalism and disordered eating is fully mediated by acculturative stress. The final best-fitting model (Model 3; Figure 2B) accounted for 58% of the variance in disordered eating in Asian American women.

## Discussion

This was the first known study to simultaneously examine sociocultural and culture-specific predictors of disordered eating in a large sample of Asian American college women and yielded some important information. First, this study replicated prior research (Nouri et al., 2011; Smart and Tsong, 2014) by demonstrating that the well-established sociocultural model for disordered eating (Thompson and Stice, 2001; Cafri et al., 2005) applies to Asian American women. Specifically, bivariate correlations and path analyses indicated that pressures for thinness and thin-ideal internalization were positively associated with disordered eating, and thin-ideal internalization partially mediated the relationship between pressures for thinness and disordered eating. These findings highlight that Asian American women are not immune to the detrimental effects of mainstream Western beauty ideals, contrary to the “model minority” stereotype (Osajima, 2005). As such, key leading factors identified in European and European American samples (Soh and Walter, 2013) are highly relevant for understanding disordered eating in Asian American women and contribute to understanding of disordered eating risk across cultures.

Second, this study suggests that culture-specific factors may be critical to eating pathology in Asian American women, above and beyond the sociocultural model. Path analyses showed that acculturative stress was a positive predictor, whereas biculturalism was an inverse predictor of disordered eating. Moreover, the relationship between biculturalism and acculturative stress was fully accounted for by acculturative stress. Given that acculturative stress fully mediated the relationship between biculturalism and disordered eating, the findings likely indicate that it is not low biculturalism alone, but low biculturalism in the face of elevated acculturative stress that contributes to disordered eating. These findings suggest that the simultaneous assessment of both biculturalism and acculturative stress is likely important, and the continued exploration of biculturalism, as opposed to unidimensional approaches (e.g., assimilation), may be useful for elucidating acculturative processes in disordered eating.

Surprisingly, contrary to hypotheses and extant findings in women of color (e.g., Rakhkovskaya and Warren, 2014, 2016), ethnic identity was not significantly associated with any of the study variables, including other culture-specific factors (e.g., biculturalism). One possible explanation is that, in contrast to other ethnic minority groups (e.g., Henrickson et al., 2010; Stein et al., 2010), many Asian/Asian American cultures value the thin ideal (Luo et al., 2005). Given that both Asian American and mainstream US cultures value the thin ideal, Asian American women may report similar pressures for thinness and thin-ideal internalization, regardless of how strongly they identify with their culture (i.e., ethnic identity). In line with this possibility, *post hoc* analyses demonstrated that participants with low vs. high ethnic identity did not significantly differ on mean levels of pressures for thinness or thin-ideal internalization [*F*(1,426) = 0.74, *p* = 0.39; *F*(1,428) = 0.50, *p* = 0.48, respectively; data available upon request].

Notably, this inconsistency with prior literature (e.g., Rakhkovskaya and Warren, 2016) does *not* appear to be explained by between-study differences in the disordered eating outcome variable (EDE-Q global score vs. weight and shape concerns subscales), as *post hoc* analyses confirmed that ethnic identity is also not significantly correlated with the weight and shape concerns subscales in this sample [*r*s = −0.06 to −0.02, *p*s = 0.20–0.57; data available upon request]. Overall, while these follow-up results are consistent with research on thin idealization in Asian and Asian American cultures (Luo et al., 2005), the differences in results between this study and others (Rakhkovskaya and Warren, 2016) are notable and could speak to sample differences. For example, in contrast to Rakhkovskaya and Warren (2016), this study included multiracial participants (although participants did not significantly differ by racial identification on study variables) and recruitment materials directly advertised to Asian American women (rather than using a general advertisement to college women). Moving forward, it will be important to further elucidate the relationship between disordered eating, sociocultural effects, and ethnic identity in Asian American women and to better understand factors that may contribute to mixed findings.

Overall, these results add to a growing literature on sociocultural predictors for disordered eating in Asian American women (Lai et al., 2013; Omori et al., 2016; Rakhkovskaya and Warren, 2016) and further highlight the additional role of key culture-specific factors – biculturalism and acculturative stress. Clinically, assessment of biculturalism and acculturative stress may be important, as these factors may contribute to symptom maintenance and may be relevant to eating disorder treatment prognosis in Asian American women. Awareness of the “model minority” stereotype (Lee et al., 2009) and how it may impact how Asian American women present to treatment and providers’ assessment of symptom severity is also important. Further, our data suggest that providers should consider Asian American women’s experience as an ethnic minority group, exposed to mainstream beauty ideals that are largely unattainable. Acculturative difficulties may make these experiences even more challenging for Asian American women, by contributing to other mental health concerns (Oh et al., 2002), as well as attenuating the protective effects of biculturalism on disordered eating. Overall, findings highlight the need for clinical practice grounded in cultural competency and humility.

### Limitations and Future Directions

This was a correlational study, and thus, findings from path analysis models cannot be used to make causal inferences. Accordingly, future studies should build on this correlational study by implementing longitudinal and experimental designs, in order to help establish causality among the predicted relationships. In addition, these findings are *not* necessarily generalizable to (1) Asian women, (2) Asian American women from ethnic groups absent from the sample (e.g., Bhutanese American women), (3) Asian Americans of other genders and age groups, (4) women of Asian descent in other diasporas (e.g., Asian Australian women), and (5) other ethnic minority women in the United States. Furthermore, participants lived in a large Southwestern metropolitan area with a unique appearance-focused micro-culture. This may result in elevated symptoms of disordered eating and sociocultural factors for women.

Replication of this model in other women of color is a critical next step, and if findings are unique for Asian American women, it would be necessary to determine why that is the case. A number of factors of interest differentiate Asian Americans from other ethnic minority groups. For example, unlike other women of color (e.g., African Americans; Latina Americans), Asian American women (1) value a thinner body ideal (Luo et al., 2005) and (2) are widely subject to the “model minority” stereotype (Osajima, 2005; Lee et al., 2009). These additional factors may account for increased rates of disordered eating in Asian American women, relative to other ethnic minority groups.

In addition, this study’s participants were heterogeneous in regard to ethnic group membership: one-third was multiracial and almost one-fifth were multi-ethnic. Furthermore, participants endorsed membership of 16 different ethnic groups, spanning South, East, and Southeast Asia, as well as Hawaii. Although we aimed to ensure that differences in generational status and racial identification (i.e., multiracial vs. uniracial Asian American) did not unduly impact our findings, important differences could exist between women with different regions of origin (e.g., South Asia vs. East Asia vs. Southeast Asia). It is also unknown how identifying with Hawaiian diaspora/origins affects Asian American women’s cultural values or experiences in the United States. Additionally, Asian American women could differ in symptomatology due to socioeconomic factors. For example, Asian or Asian American women may experience differences in exposure to and/or reactivity to body ideals based on whether one has been raised in urban/developed vs. rural/underdeveloped areas, or whether one has immigrated for better economic opportunities vs. fleeing poverty or dangerous conditions. Examination of within-group differences among Asian American women is a critical next step.

## Conclusion

This study highlights that Asian American women are not immune to the detrimental effects of mainstream Western media, contrary to the “model minority” stereotype. Key leading factors identified in European and European American samples (i.e., thin-ideal internalization and pressures for thinness) are highly relevant for understanding disordered eating in Asian American women. Furthermore, this study highlights how Asian American women’s experiences within a minority culture may result in unique mental health difficulties (i.e., heightened disordered eating symptoms) that tend to be less prevalent among other ethnic minority groups. Finally, culture-specific factors, such as acculturative stress and biculturalism, may play a role in elucidating disordered eating risk in Asian American women. Findings highlight the importance of focusing on sociocultural factors as key players of disordered eating, while also remaining open to the likely relevance of culture-specific factors, such as acculturative stress and biculturalism.

## Data Availability

The datasets generated for this study are available on request to the corresponding author.

## Ethics Statement

Human Subject Research: The studies involving human participants were reviewed and approved by the University of Nevada, Las Vegas Institutional Review Board. The participants provided their written informed consent to participate in this study.

## Author Contributions

LA and KC designed the study and wrote the protocol. LA completed the literature search, collected the data, conducted the statistical analyses, and drafted the manuscript. KC oversaw the data collection. KC and CW edited the manuscript. All authors approved the final version of the manuscript.

## Conflict of Interest Statement

CW is employed by the Choose Honesty, LLC. The remaining authors declare that the research was conducted in the absence of any commercial or financial relationships that could be construed as a potential conflict of interest.
